# Wheat Transcription Factor TaMYB60 Modulates Cuticular Wax Biosynthesis by Activating *TaFATB* and *TaCER1* Expression

**DOI:** 10.3390/ijms251910335

**Published:** 2024-09-26

**Authors:** Xiaoyu Wang, Wanzhen Chen, Pengfei Zhi, Cheng Chang

**Affiliations:** College of Life Sciences, Qingdao University, Qingdao 266071, China

**Keywords:** wheat, MYB transcription factor, cuticular wax biosynthesis, fatty acyl-ACP thioesterases, *ECERIFERUM 1*

## Abstract

Cuticular wax mixtures cover the epidermis of land plants and shield plant tissues from abiotic and biotic stresses. Although cuticular wax-associated traits are employed to improve the production of bread wheat, regulatory mechanisms underlying wheat cuticular wax biosynthesis remain poorly understood. In this research, partially redundant transcription factors TaMYB60-1 and TaMYB60-2 were identified as positive regulators of wheat cuticular wax biosynthesis. Knock-down of wheat *TaMYB60-1* and *TaMYB60-2* genes by virus-induced gene silencing resulted in attenuated wax accumulation and enhanced cuticle permeability. The roles of wheat fatty acyl-ACP thioesterase genes *TaFATB1* and *TaFATB2* in cuticular wax biosynthesis were characterized. Silencing wheat *TaFATB1* and *TaFATB2* genes led to reduced wax accumulation and increased cuticle permeability, suggesting that *TaFATB1* and *TaFATB2* genes positively contribute to wheat cuticular wax biosynthesis. Importantly, transcription factors TaMYB60-1 and TaMYB60-2 exhibit transcriptional activation ability and could stimulate the expression of wax biosynthesis genes *TaFATB1*, *TaFATB2*, and *ECERIFERUM 1* (*TaCER1*). These findings support that transcription factor TaMYB60 positively regulates wheat cuticular wax biosynthesis probably by activating transcription of *TaFATB1*, *TaFATB2*, and *TaCER1* genes.

## 1. Introduction

The lipophilic cuticle covers plant aerial and underground portions, including nonwoody stems, leaves, flowers, and root tips, during their primary growth [1,2,3,4,5,6,7]. Through shielding plant tissues from challenging environments, protective cuticles greatly contribute to plant adaptation to abiotic and biotic stresses like water deficit (drought), flooding, ultraviolet (UV) radiation, unfavorable temperatures, and attacks from pathogens and pests (P&Ps) [8,9,10,11,12]. In addition, many developmental events like organ separation and later root formation are governed by the cuticle [2,4,13,14]. Although chemical compositions and amounts of the plant cuticle depend on plant species, organs, plant developmental stages, and environmental conditions, the cuticle mainly consists of cutin polyester matrices covered and sealed with cuticular wax mixtures [15,16].

Cuticular wax mixtures are organic solvent-extractable constituents and are mainly composed of very long-chain (VLC, >C20) fatty acids and their derivatives like alkanes, primary and secondary alcohols, aldehydes, ketones, alkenes, and esters [17,18,19,20,21]. Cuticular wax biosynthesis has been extensively studied in the dicot model plant *Arabidopsis thaliana* through genetic screening of *Arabidopsis eceriferum* (*cer*) mutants and chemical analysis of cuticular wax composition by gas chromatography (GC) [17,18,19,20,21]. Briefly, C16 and C18 fatty acyl–acyl carrier proteins (ACPs) generated by de novo fatty acid synthesis in plastids are hydrolyzed by acyl-ACP thioesterases (FATB) to release free C16 and C18 fatty acids and are then exported to the cytosol [22]. Cuticular wax biosynthesis mainly occurs in the endoplasmic reticulum (ER), where the saturated C16 and C18 fatty acids are firstly esterified to fatty acyl-CoA by long-chain acyl-coenzyme A synthases (LACS) [23,24,25]. Under the action of the fatty acid elongase (FAE) complex, C16 and C18 fatty acyl-CoA precursors are then elongated to VLC acyl-CoAs [26,27,28,29,30,31,32,33,34]. These VLC acyl-CoAs could be either converted to VLC aldehydes, alkanes, secondary alcohols, and ketones via the alkane-forming pathway catalyzed by the CER1/CER3/CYTOCHROME B5 (CYTB5) complex and CYP95A family cytochrome P450 enzymes MIDCHAIN ALKANE HYDROXYLASE1 (MAH1), or modified to VLC primary alcohols and esters via the alcohol-forming pathway catalyzed by the CER1/CER3/CYTOCHROME B5 (CYTB5) complex [35,36,37,38,39,40,41,42,43]. These cuticular wax constituents are then exported out of the ER and finally deposited to the extracellular cuticular regions by the trans-Golgi network (TGN) trafficking pathways, ATP binding cassette (ABC) transporters, as well as lipid transfer proteins (LTPs) [44,45,46,47,48,49,50,51,52,53,54,55]. While the cuticular wax biosynthetic pathway is well documented in the dicot model plant *Arabidopsis*, the molecular mechanism underlying cuticular wax biosynthesis is not well understood in the agronomically important cereal crop bread wheat.

Increasing evidence revealed that cuticular wax biosynthesis is regulated at the transcriptional level in model and crop plants [18,19,20]. For instance, *Arabidopsis* transcription factors AtMYB16, AtMYB30, AtMYB94, AtMYB96, and AtMYB106 tightly regulate cuticular wax biosynthesis in response to developmental and environmental cues [56,57,58,59,60,61,62,63]. *Arabidopsis* transcription factors SHINE1/WAXINDUCER1 (AtSHN1/WIN1) and DEWAX were identified, respectively, as positive and negative regulators of cuticular wax biosynthesis [64,65,66,67,68,69]. Similarly, wheat AP2/ERF-type transcription factor TaSHN1/WIN1, basic helix-loop-helix (bHLH)-type transcription factor, *TaKCS6* promoter-associated bHLH type transcription factor 1 (TaKPAB1), MYB-type transcription factors TaMYB30 and TaMIXTA1/2 and *TaECR* promoter-binding MYB transcription factor 1 (TaEPBM1)/MYB96 positively regulate cuticular wax biosynthesis [70,71,72,73,74,75]. In rice, AP2/ERF-type transcription factor wax synthesis regulatory gene 1 (OsWR1) and OsWR2, as well as MYB-type transcription factor OsMYB60, were identified as activators of cuticular wax biosynthesis [76,77]. However, whether and how wheat homologs of MYB60 become involved in the regulation of cuticular wax biosynthesis remains to be disclosed.

In this study, partially redundant transcription factors TaMYB60-1 and TaMYB60-2 were identified as positive regulators of wheat cuticular wax biosynthesis. Silencing of wheat *TaMYB60-1* and *TaMYB60-2* genes resulted in attenuated wax accumulation and potentiated cuticle permeability. Furthermore, wheat fatty acyl-ACP thioesterase genes *TaFATB1* and *TaFATB2* were found to be essential for cuticular wax accumulation. Silencing wheat *TaFATB1* and *TaFATB2* genes by virus-induced gene silencing led to reduced wax accumulation and enhanced cuticle permeability. Importantly, transcription factors TaMYB60-1 and TaMYB60-2 exhibit transcriptional activation ability and could directly activate the expression of wax biosynthesis genes *TaFATB1*, *TaFATB2*, and *TaCER1*. This evidence supports the idea that transcription factor TaMYB60 positively regulates wheat cuticular wax biosynthesis probably by activating transcription of *TaFATB1*, *TaFATB2* and *TaCER1* genes.

## 2. Results

### 2.1. Identification of Wheat TaMYB60-1 and TaMYB60-2 Based on Homology with Arabidopsis AtMYB60 and Rice OsMYB60 Proteins

**Figure 1:**
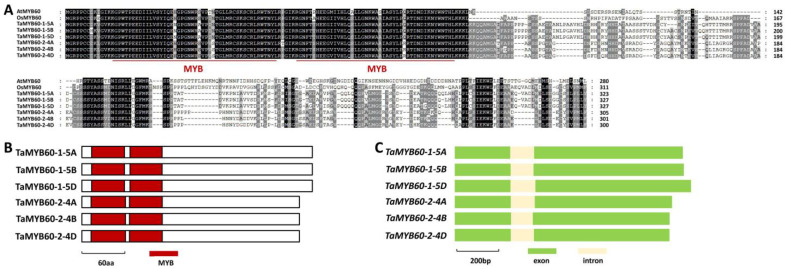
Homology-based identification of wheat transcription factors TaMYB60-1 and TaMYB60-2. (**A**) Protein sequence comparison of wheat MYB transcription factors TaMYB60-1-5A, TaMYB60-1-5B, TaMYB60-1-5D, TaMYB60-2-4A, TaMYB60-2-4B, TaMYB60-2-4D, *Arabidopsis* AtMYB60 and rice OsMYB60. Residue numbers were labeled. Identical residues among 8 protein sequences are shaded in black, while residues conserved in at least 4 of the 8 proteins are shaded in gray. (**B**) Domain structures of wheat TaMYB60-1 and TaMYB60-2 proteins. (**C**) Gene architectures of wheat *TaMYB60-1* and *TaMYB60-2* genes.

Rice MYB-type transcription factor OsMYB60, a homolog of *Arabidopsis* AtMYB60, positively contributes to rice wax biosynthesis, but wheat homologs of MYB60 remain to be identified [77]. In this research, we employed *Arabidopsis* AtMYB60 (At1g08810) and rice OsMYB60 (LOC_Os12g03150) to search the reference genome of allohexaploid bread wheat. Wheat TaMYB60-1 and TaMYB60-2 were identified as the closed homologs of *Arabidopsis* AtMYB60 and rice OsMYB60 (Figure S1). As shown in Figure 1A, *TaMYB60-1-5A* (*TraesCS5A02G142500*), *TaMYB60-1-5B* (*TraesCS5B02G141200*), and *TaMYB60-1-5D* (*TraesCS5D02G149600*) located on chromosomes 5A, 5B, and 5D of allohexaploid bread wheat, respectively, are three highly homologous sequences of the *TaMYB60-1* gene. Similarly, three highly homologous sequences of *TaMYB60-2* genes were obtained from wheat chromosomes 4A, 4B, and 4D and designated as *TaMYB60-2-4A* (*TraesCS4A02G189700*), *TaMYB60-2-4B* (*TraesCS4B02G129100*) and *TaMYB60-2-4D* (*TraesCS4D02G123300*), respectively.

As shown in Figure 1A, these predicted amino acid sequences of TaMYB60-1-5A, TaMYB60-1-5B, TaMYB60-1-5D, TaMYB60-2-4A, TaMYB60-2-4B, and TaMYB60-2-4D proteins shared more than 43% of their identities with *Arabidopsis* AtMYB60 and rice OsMYB60 proteins. Two MYB motifs were identified from the N-terminal parts of TaMYB60-1-5A, TaMYB60-1-5B, TaMYB60-1-5D, TaMYB60-2-4A, TaMYB60-2-4B, and TaMYB60-2-4D proteins (Figure 1B). Coding regions of *TaMYB60-1-5A*, *TaMYB60-1-5B*, *TaMYB60-1-5D*, *TaMYB60-2-4A*, *TaMYB60-2-4B*, and *TaMYB60-2-4D* genes all contain two exons and one intron (Figure 1C).

### 2.2. Partially Redundant TaMYB60-1 and TaMYB60-2 Transcription Factors Positively Regulate Wheat Cuticular Wax Biosynthesis

**Figure 2:**
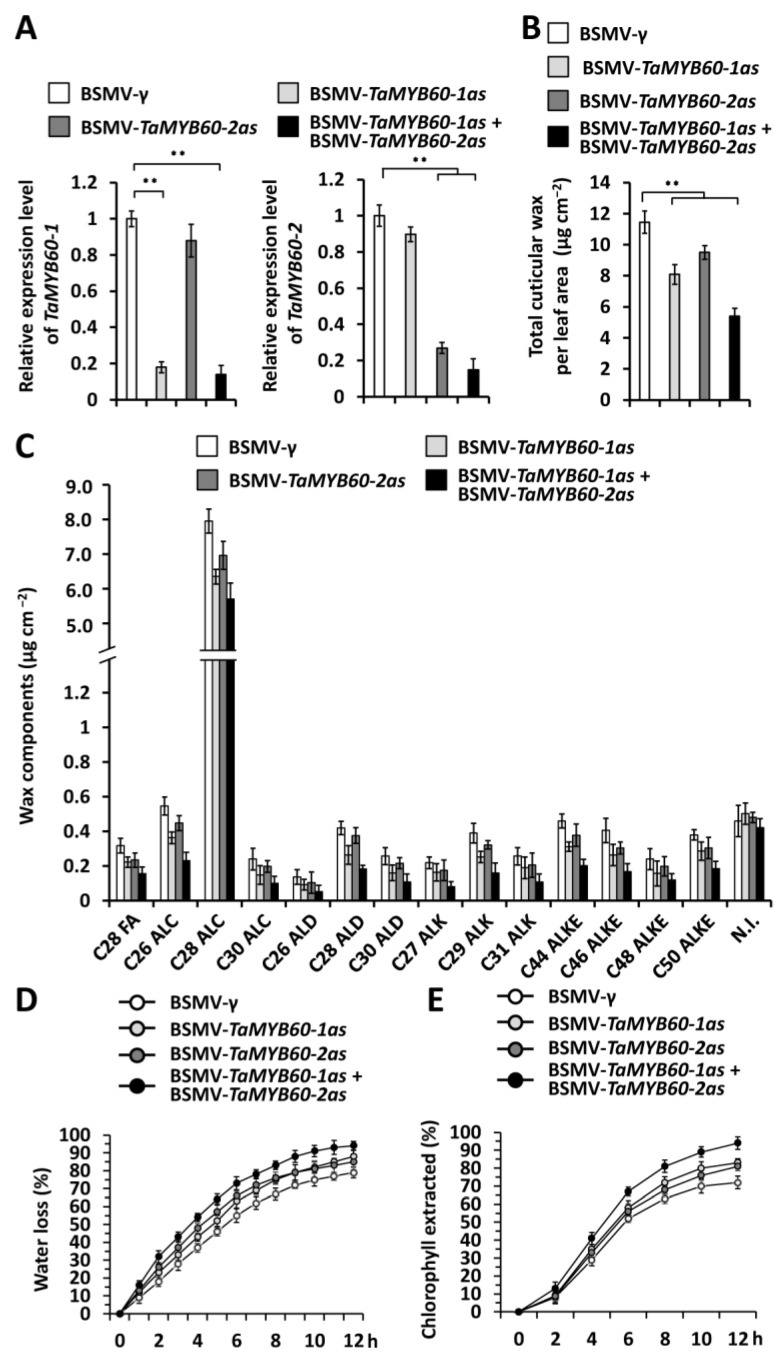
Functional analyses of *TaMYB60-1* and *TaMYB60-2* genes in wheat cuticular wax accumulation. (**A**) qRT-PCR analysis of *TaMYB60-1* and *TaMYB60-2* expression levels in the wheat leaves silencing *TaMYB60-1* (BSMV-*TaMYB60-1as*), *TaMYB60-2* (BSMV-*TaMYB60-2as*), or co-silencing *TaMYB60-1* and *TaMYB60-2* (BSMV-*TaMYB60-1as* + BSMV-*TaMYB60-2as*). (**B**) Total cuticular wax amounts in the wheat leaves silencing *TaMYB60-1*, *TaMYB60-2*, or co-silencing *TaMYB60-1* and *TaMYB60-2*. (**C**) Amounts of major cuticular wax components in the wheat leaves silencing *TaMYB60-1*, *TaMYB60-2*, or co-silencing *TaMYB60-1* and *TaMYB60-2*. FA, fatty acid; ALC, alcohol; ALD, aldehyde; ALK, alkane; ALKE, alkyl ester; N.I., not identified compound. (**D**) Water loss rates and (**E**) chlorophyll extraction levels analyzed in wheat leaves *TaMYB60-1*, *TaMYB60-2*, or co-silencing *TaMYB60-1* and *TaMYB60-2.* For (**A**,**B**), three biological replicates were statistically analyzed for each treatment, and data are presented as the mean ± SE (Student’s *t*-test, ** *p* < 0.01).

To examine the potential regulation of *TaMYB60-1* and *TaMYB60-2* on wheat cuticular wax biosynthesis, we conducted the barley stripe mosaic virus-induced gene silencing (BSMV-VIGS) assay to separately silence all endogenous *TaMYB60-1* or *TaMYB60-2* genes in the wheat cultivar Yannong 999. It was previously demonstrated that BSMV infection did not change the wax deposition and composition significantly [74]. Quantitative reverse transcription-polymerase chain reaction (qRT-PCR) assay validated that levels of *TaMYB60-1* or *TaMYB60-2* gene transcripts were remarkably reduced in wheat leaves silencing *TaMYB60-1*, *TaMYB60-2*, or co-silencing *TaMYB60-1* and *TaMYB60-2* (Figure 2A). After that, we employed gas chromatography–mass spectrometry (GC–MS) to analyze the cuticular wax mixtures in the wheat leaves silencing *TaMYB60-1*, *TaMYB60-2*, or co-silencing *TaMYB60-1* and *TaMYB60-2*. As shown in Figure 2B, the amount of cuticular wax accumulated on wheat leaves decreased significantly from 11.45 μg cm^−2^ in the BSMV-*γ*-infected plants to below 9.56 μg cm^−2^ in the wheat plants silencing *TaMYB60-1* or *TaMYB60-2*. In addition, co-silencing *TaMYB60-1* and *TaMYB60-2* genes resulted in a further reduction in cuticular wax accumulation to 5.39 μg cm^−2^ (Figure 2B). Further cuticular wax composition analyses revealed that major cuticular wax compositions like VLC alcohols, alkanes, aldehydes, and esters showed a decrease to a different extent in the wheat leaves silencing *TaMYB60-1*, *TaMYB60-2*, or co-silencing *TaMYB60-1* and *TaMYB60-2*, compared with the BSMV-*γ* control (Figure 2C). Excised-leaf water loss and chlorophyll leaching were then measured to analyze the potential cuticle permeability regulation of TaMYB60. As shown in Figure 2D,E, enhanced water loss and chlorophyll leaching was observed on wheat leaves expressing silencing constructs for TaMYB60-1 or TaMYB60-2, compared with the BSMV-*γ* control. Co-silencing *TaMYB60-1* and *TaMYB60-2* genes resulted in further increased water loss and chlorophyll leaching (Figure 2D,E). These results collectively suggest that partially redundant *TaMYB60-1* and *TaMYB60-2* genes positively regulate wheat cuticular wax accumulation and that this contributes to barrier properties of the cuticle.

### 2.3. Identification of Wheat TaFATB1 and TaFATB2 Based on Homology with Arabidopsis AtFATB Protein

**Figure 3:**
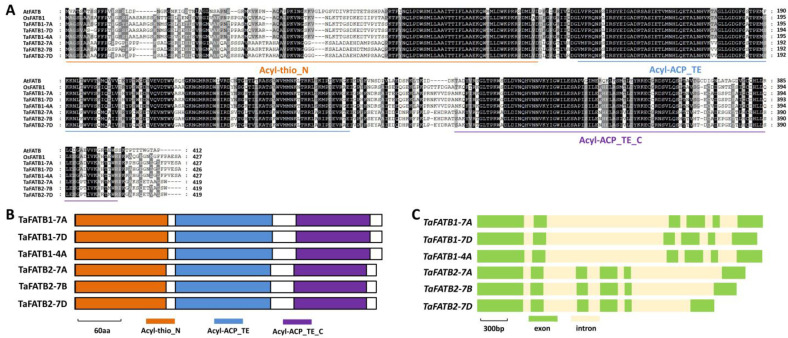
Homology-based identification of wheat fatty acyl-ACP thioesterases TaFATB1 and TaFATB2. (**A**) Protein sequence comparison of wheat fatty acyl-ACP thioesterases TaFATB1-7A, TaFATB1-7B, TaFATB1-7D, TaFATB2-7A, TaFATB2-7B, TaFATB2-7D, *Arabidopsis* AtFATB and rice OsFATB1. Residue numbers were labeled. Identical residues among 8 protein sequences are shaded in dark, while residues conserved in at least 4 of the 8 proteins are shaded in gray. (**B**) Domain structures of wheat TaFATB1 and TaFATB2 proteins. (**C**) Gene architectures of wheat *TaFATB1* and *TaFATB2* genes.

*Arabidopsis* fatty acyl-ACP thioesterase *AtFATB* plays an essential role in cuticular wax biosynthesis, but wheat homologs of FATB remain to be identified [22]. In this research, we employed *Arabidopsis* AtFATB (At1g08510) as a query to search the reference genome of allohexaploid bread wheat. Wheat TaFATB1 and TaFATB2 were identified as the closed homologs of *Arabidopsis* AtFATB (Figure S2). As shown in Figure 3A, *TaFATB1-7A* (*TraesCS7A02G089000*), *TaFATB1-7D* (*TraesCS7D02G084400*) and *TaFATB1-4A* (*TraesCS4A02G387700*) located on chromosomes 7A, 7D and 4A of allohexaploid bread wheat, respectively, are three highly homologous sequences of the *TaFATB1* gene. Similarly, three highly homologous sequences of *TaFATB2* genes were obtained from wheat chromosomes 7A, 7B, and 7D and designated as *TaFATB2-7A* (*TraesCS7A02G371900*), *TaFATB2-7B* (*TraesCS7B02G255800*) and *TaFATB2-7D* (*TraesCS7D02G351000*), respectively.

As shown in Figure 3A, these predicted amino acid sequences of TaFATB1-7A, TaFATB1-7D, TaFATB1-4A, TaFATB2-7A, TaFATB2-7B, and TaFATB2-7D proteins shared more than 55% of their identities with *Arabidopsis* AtFATB protein. Acyl-ATP thioesterase (Acyl-thio_N), Acyl-ATP thioesterase N-terminal domain (Acyl-ACP_TE) and Acyl-ATP thioesterase C-terminal domain (Acyl-ACP_TE_C) were identified from the N-terminal, middle and C-terminal parts of all TaFATB proteins, respectively (Figure 3B). Coding regions of *TaFATB1-7A*, *TaFATB1-7D*, *TaFATB1-4A*, *TaFATB2-7A*, *TaFATB2-7B* and *TaFATB2-7D* genes all contain six exons and five introns (Figure 3C).

### 2.4. Wheat TaFATB1 and TaFATB2 Genes Positively Contribute to Wheat Cuticular Wax Accumulation

**Figure 4:**
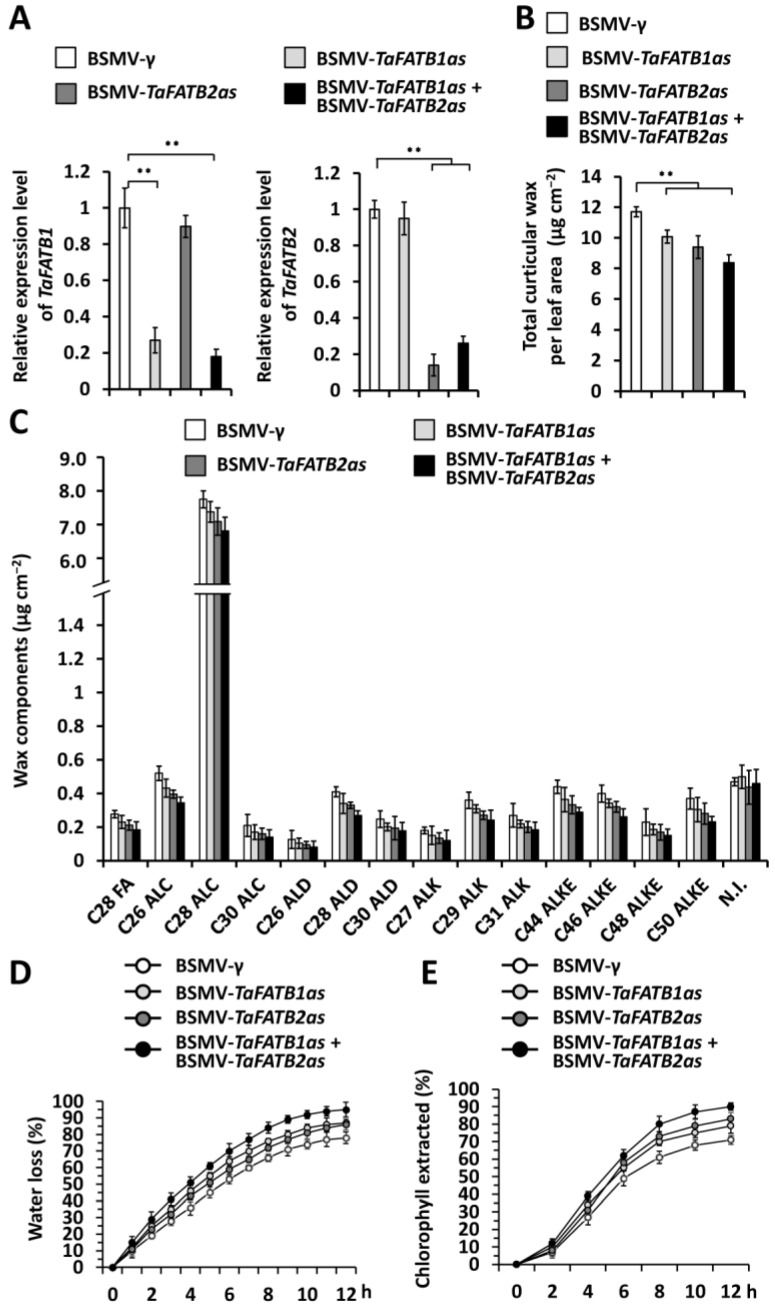
Functional analyses of wheat *TaFATB1* and *TaFATB2* genes in wheat cuticular wax accumulation. (**A**) qRT-PCR analysis of *TaFATB1* and *TaFATB2* expression levels in the wheat leaves silencing *TaFATB1* (BSMV-*TaFATB1as*), *TaFATB2* (BSMV-*TaFATB2as*), or co-silencing *TaFATB1* and *TaFATB2* (BSMV-*TaFATB1as* + BSMV-*TaFATB2as*). (**B**) Total cuticular wax amounts in the wheat leaves silencing *TaFATB1*, *TaFATB2*, or co-silencing *TaFATB1* and *TaFATB2*. (**C**) Amounts of major cuticular wax components in the wheat leaves silencing *TaFATB1*, *TaFATB2*, or co-silencing *TaFATB1* and *TaFATB2*. (**D**) Water loss rates and (**E**) chlorophyll extraction levels analyzed in wheat leaves *TaFATB1*, *TaFATB2*, or co-silencing *TaFATB1* and *TaFATB2.* For (**A**,**B**), three biological replicates were statistically analyzed for each treatment, and data are presented as the mean ± SE (Student’s *t*-test, ** *p* < 0.01).

To examine the potential contribution of *TaFATB1* and *TaFATB2* to wheat cuticular wax biosynthesis, we performed BSMV-VIGS to separately silence all endogenous *TaFATB1* or *TaFATB2* genes in the wheat cultivar Yannong 999. qRT-PCR assay confirmed that levels of *TaFATB1* or *TaFATB2* gene transcripts were remarkably reduced in wheat leaves silencing *TaFATB1*, *TaFATB2*, or co-silencing *TaFATB1* and *TaFATB2* (Figure 4A). Thereafter, we employed GC–MS to analyze the cuticular wax mixtures in the wheat leaves silencing *TaFATB1*, *TaFATB2*, or co-silencing *TaFATB1* and *TaFATB2*. As shown in Figure 4B, the amount of cuticular wax accumulated on wheat leaves decreased significantly from 11.71 μg cm^−2^ in the BSMV-*γ*-infected plants to below 10.09 μg cm^−2^ in the wheat plants silencing *TaFATB1* or *TaFATB2*. In addition, co-silencing *TaFATB1* and *TaFATB2* genes resulted in a further reduction in cuticular wax accumulation to 8.46 μg cm^−2^ (Figure 4B). Further cuticular wax composition analyses revealed that major cuticular wax compositions like VLC alcohols, alkanes, aldehydes, and esters showed a decrease to a different extent in the wheat leaves silencing *TaFATB1*, *TaFATB2*, or co-silencing *TaFATB1* and *TaFATB2*, compared with the BSMV-*γ* control (Figure 4C). Excised-leaf water loss and chlorophyll leaching were then measured to analyze the potential regulation of *TaFATB* on cuticle permeability. As shown in Figure 4D,E, enhanced water loss and chlorophyll leaching were observed on the wheat leaves silencing *TaFATB1* or *TaFATB2*, compared with the BSMV-*γ* control. Co-silencing *TaFATB1* and *TaFATB2* genes resulted in further increased water loss and chlorophyll leaching (Figure 4D,E). These results collectively suggested that partially redundant *TaFATB1* and *TaFATB2* positively contribute to wheat cuticular wax accumulation.

### 2.5. Transcriptional Activators TaMYB60-1 and TaMYB60-2 Directly Activate Transcription of TaFATB1, TaFATB2 and TaCER1 Genes

**Figure 5:**
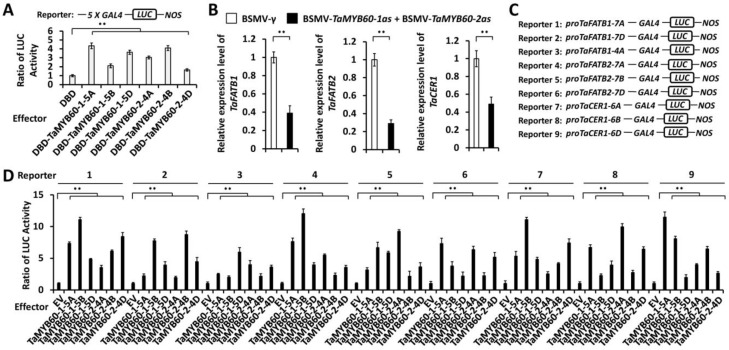
Analysis of the transcriptional regulation of *TaFATB1*, *TaFATB2*, and *TaCER1* genes by wheat transcriptional activators TaMYB60-1 and TaMYB60-2. (**A**) Transcriptional activation ability of wheat transcription factors TaMYB60-1 and TaMYB60-2 measured in *Arabidopsis* protoplast cells. (**B**) Expression levels of *TaFATB1*, *TaFATB2*, and *TaCER1* in the wheat leaves co-silencing *TaMYB60-1* and *TaMYB60-2* were measured with qRT-PCR assay. (**C**) Schematic depiction of the LUCIFERASE (LUC) reporter containing promoter fragments of *TaFATB1-7A*, *TaFATB1-7B*, *TaFATB1-7D*, *TaFATB2-7A*, *TaFATB2-7B*, *TaFATB2-7D*, *TaCER1-6A*, *TaCER1-6B*, or *TaCER1-6D* genes. Schematic for the 9 constructs relates to the experiment in Figure 5D. (**D**) Activation of *TaFATB1*, *TaFATB2*, or *TaCER1* promoters by wheat transcription factors TaMYB60-1 and TaMYB60-2 in *Arabidopsis* protoplast cells. For (**A**,**B**,**D**), three biological replicates were statistically analyzed for each treatment, and data are presented as the mean ± SE (Student’s *t*-test, ** *p* < 0.01).

Rice MYB-type transcription factor OsMYB60 functions as a transcriptional activator [77]. We performed the dual-luciferase reporter assay using the *Arabidopsis* protoplast system to measure the transcriptional activation activity of TaMYB60-1 and TaMYB60-2 proteins. As shown in Figure 5A, the overaccumulation of effector proteins DBD-TaMYB60-1-5A, DBD-TaMYB60-1-5B, DBD TaMYB60-1-5D, DBD-TaMYB60-2-4A, DBD-TaMYB60-2-4B, or DBD-TaMYB60-2 4D resulted in the reporter luciferase activity (LucA) ratio being increased to above 1.71 from 1 for the DBD control. Similarly, overaccumulation of effector protein DBD-AtMYB60 led an increased LucA ratio of 2.63 (Figure S3). This result indicates that TaMYB60-1-5A, TaMYB60-1-5B, TaMYB60-1-5D, TaMYB60-2-4A, TaMYB60-2-4B and TaMYB60-2-4D proteins, resembling AtMYB60, exhibit transcriptional activation activity in plant cells.

Previous studies showed that expressions of cuticular wax biosynthesis genes *OsCER1* and *OsFATB1* were reduced in the rice *osmyb60* mutant compared with the wild type plants [77]. It has been demonstrated that TaCER1 genes contribute to wheat cuticular wax biosynthesis [78]. To examine the potential regulation of partially redundant TaMYB60-1 and TaMYB60-2 on the transcription of *TaFATB1*, *TaFATB2* and *TaCER1*, we analyzed the expression levels of *TaFATB1*, *TaFATB2* and *TaCER1* genes in the wheat leaves co-silenced with *TaMYB60-1* and *TaMYB60-2* constructs. As shown in Figure 5B, qRT-PCR assay results demonstrated that levels of *TaFATB1*, *TaFATB2*, and *TaCER1* transcripts were significantly reduced in wheat leaves co-silenced with *TaMYB60-1* and *TaMYB60-2* constructs compared with the control BSMV-*γ* leaves, suggesting that partially redundant TaMYB60-1 and TaMYB60-2 positively regulate the expressions of *TaFATB1*, *TaFATB2*, and *TaCER1* genes. Thereafter, we performed the dual-luciferase reporter assay to examine the potential transactivation of *TaFATB1*, *TaFATB2*, and *TaCER1* promoters by transcriptional activators TaMYB60-1 and TaMYB60-2. LUC reporters harboring promoter regions of *TaFATB1-7A*, *TaFATB1-7D*, *TaFATB1-4A*, *TaFATB2-7A*, *TaFATB2-7B*, *TaFATB2-7D*, *TaCER1-6A*, *TaCER1-6B*, and *TaCER1-6D* genes were co-expressed with effector proteins TaMYB60-1-5A, TaMYB60-1-5B, TaMYB60-1-5D, TaMYB60-2-4A, TaMYB60-2-4B, or TaMYB60-2-4D (Figure 5C and Figure S4). As shown in Figure 5D, overaccumulation of effector proteins TaMYB60-1-5A, TaMYB60-1-5B, TaMYB60-1-5D, TaMYB60-2-4A, TaMYB60-2-4B, or TaMYB60-2-4D resulted in the LucA ratio increasing to above 1.93 from 1 for the empty vector (EV) control, suggesting that wheat transcriptional activators TaMYB60-1 and TaMYB60-2 could directly activate promoters of *TaFATB1*, *TaFATB2*, and *TaCER1* genes. The evidence presented strongly supports that transcriptional activators *TaMYB60-1* and *TaMYB60-2* directly stimulate expression of cuticular wax biosynthesis genes *TaFATB1*, *TaFATB2* and *TaCER1* to positively regulate wheat cuticular wax accumulation.

## 3. Discussion

### 3.1. Wheat MYB Transcription Factors TaMYB60-1 and TaMYB60-2 Are Key Regulators of Cuticular Wax Biosynthesis

In this study, MYB transcription factors TaMYB60-1 and TaMYB60-2 were identified as positive regulators of wheat cuticular wax biosynthesis. Knock-down of wheat *TaMYB60-1* or *TaMYB60-2* gene by virus-induced gene silencing resulted in attenuated wax accumulation and potentiated leaf water loss and chlorophyll leaching. Notably, co-silencing *TaMYB60-1* and *TaMYB60-2* genes resulted in a further reduction in cuticular wax accumulation, as well as a further increase in cuticle permeability, indicating that partially redundant TaMYB60-1 and TaMYB60-2 transcription factors positively regulate wheat cuticular wax biosynthesis. Previous research showed that rice *osmyb60* null mutants (*osmyb60-1* and *osmyb60-2*) exhibited reduced cuticular wax, as well as increased water loss rate and the chlorophyll leaching ratio, compared with the wild type plant, indicating that rice transcription factor OsMYB60 stimulates cuticular wax accumulation [77]. These results suggest that positive regulation of cuticular wax biosynthesis by transcription factor MYB60 might be conserved among rice and bread wheat. Indeed, accumulating evidence revealed that regulation of some transcription factors on cuticular wax biosynthesis is highly conserved. For instance, wheat transcription factors TaMYB30, TaMIXTA1, TaMIXTA2, TaEPBM1/TaMYB96, and TaSHN1/TaWIN1, resembling their orthologs in the dicot model plant *A. thaliana*, positively regulate cuticular wax accumulation [70,71,72,73,74,75].

### 3.2. Wheat Fatty Acyl-ACP Thioesterases TaFATB1 and TaFATB2 Play Essential Roles in Wheat Cuticular Wax Biosynthesis

Wheat fatty acyl-ACP thioesterases TaFATB1 and TaFATB2 were characterized as essential components of cuticular wax biosynthetic pathways in this study. Silencing wheat genes *TaFATB1* or *TaFATB2* by BSMV-VIGS resulted in attenuated wax accumulation and potentiated leaf water loss and chlorophyll leaching. Significantly, co-silencing *TaFATB1* and *TaFATB2* genes resulted in a further reduction in cuticular wax accumulation, as well as a further increase in cuticle permeability, indicating that *TaFATB1* and *TaFATB2* partially and redundantly contribute to wheat cuticular wax biosynthesis. Previous studies in Arabidopsis showed that the total wax load of fatb-ko plants, an Arabidopsis mutant with a T-DNA insertion in the FATB gene, was reduced by 20% in leaves and by 50% in stems, implicating AtFATB in the supply of saturated fatty acids for wax biosynthesis [22]. In this study, cuticular wax composition analysis demonstrated that no novel components or substantial changes in the distribution of wax components were observed in the wheat leaves silencing TaFATB1, TaFATB2, or co-silencing TaFATB1 and TaFATB2, which is consistent with the fact that TaFATB1 and TaFATB2, resembling their ortholog in Arabidopsis, function to supply saturated fatty acids for cuticular wax biosynthesis. Indeed, studies increasingly revealed that the cuticular wax biosynthetic pathway is highly conserved between the dicot model plant A. thaliana and monocot cereal crop bread wheat. For instance, wheat TaKCS6, TaKCS1, TaCER1, TaCER5, and TaECR, resembling their Arabidopsis orthologs in Arabidopsis, function as essential components in cuticular wax biosynthesis [70,71,72,73,74,75].

### 3.3. Transcriptional Activators TaMYB60-1 and TaMYB60-2 Directly Stimulate Transcription of TaFATB1, TaFATB2, and TaCER1 Genes to Positively Regulate Wheat Cuticular Wax Biosynthesis

Previous studies demonstrated that rice MYB-type transcription factor OsMYB60 functions as a transcriptional activator and could positively regulate expression of wax biosynthesis genes *OsCER1* and *OsFATB* [77]. In this study, wheat MYB transcription factors TaMYB60-1 and TaMYB60-2, including TaMYB60-1-5A, TaMYB60-1-5B, TaMYB60-1-5D, TaMYB60-2-4A, TaMYB60-2-4B, or TaMYB60-2-4D, could activate the *LUC* reporter gene in the Arabidopsis protoplast cells, indicating that MYB transcription factors TaMYB60-1 and TaMYB60-2 possess transcriptional activation activity and could transactivate target genes in plant cells. qPCR assay results demonstrated that transcript accumulations of cuticular wax biosynthesis genes TaFATB1, TaFATB2, and TaCER1 were significantly reduced in the wheat leaves co-silencing TaFATB1 and TaFATB2. Interestingly, all TaMYB60-1 and TaMYB60-2 transcription factors like TaMYB60-1-5A, TaMYB60-1-5B, TaMYB60-1-5D, TaMYB60-2-4A, TaMYB60-2-4B, or TaMYB60-2-4D, could directly transactivate promoters of TaFATB1, TaFATB2, and TaCER1 genes, including TaFATB1-7A, TaFATB1-7D, TaFATB1-4A, TaFATB2-7A, TaFATB2-7B, TaFATB2-7D, TaCER1-6A, TaCER1-6B, and TaCER1-6D. These results suggested that transcriptional regulation of FATB and CER1 by MYB60 transcription factor essential for cuticular wax biosynthesis might be conserved among rice and bread wheat. Indeed, accumulating studies from Arabidopsis and bread wheat have revealed that transcriptional regulation of cuticular wax biosynthesis genes by MYB transcription factors is highly conserved. For instance, wheat MYB transcription factor TaEPBM1/TaMYB96, resembling Arabidopsis AtMYB96, could directly activate the expression of cuticular wax biosynthesis genes TaECR and TaCER1 to potentiate cuticular wax accumulation [74]. Another MYB transcription factor, TaMYB30, like its Arabidopsis homolog AtMYB30, could directly stimulate cuticular wax biosynthesis genes TaKCS1 and TaECR to positively regulate cuticular wax biosynthesis [71]. Wheat TaMIXTA1 and TaMIXTA2, resembling their homologs AtMYB16 and AtMYB106 from Arabidopsis, positively regulate cuticular wax accumulation via activating transcription of wax biosynthesis gene *TaKCS1* and wax deposition gene *TaCER5* [75].

In this research, wheat cuticular wax biosynthesis gene *TaCER1* was demonstrated to be directly activated by MYB transcription factors TaMYB60-1 and TaMYB60-2. Interestingly, TaCER1 was previously identified as a target gene of another MYB transcription factor TaEPBM1/TaMYB96. Indeed, increasing evidence supports that the same cuticular wax biosynthesis gene could be targeted by different transcription factors. For instance, wheat cuticular wax biosynthesis gene *TaKCS1* is directly activated by MYB transcription factors TaMYB30, TaMIXTA1, and TaMIXTA2 [71,75]. Similarly, another cuticular wax biosynthesis gene *TaECR* is targeted by MYB-type transcription factors TaEPBM1/TaMYB96 and TaMYB30 [71,74]. Therefore, it is intriguing to explore the interplay among different transcription factors targeting common cuticular wax biosynthesis genes in future research.

An increasing number of studies have revealed that transcription factors could recruit a variety of regulatory proteins, including mediators, chromatin remodelers, and histone modifying enzymes, to govern cuticular wax biosynthesis. For instance, wheat AP2/ERF-type transcription factor TaSHN1/WIN1 recruits the mediator kinase subunit TaCDK8 to potentiate cuticular wax biosynthesis [73]. bHLH-type transcription factor TaKPAB1 recruits the CHD3 protein TaCHR729 to facilitate the trimethylation of histone H3 Lys 4 (H3K4me3) at the TaKCS6 promoters, leading to the potentiated cuticular wax biosynthesis [70]. Another MYB-type transcription factor TaEPBM1/TaMYB96 was demonstrated to interact with the adapter protein Alteration/Deficiency in Activation 2 (TaADA2) and recruit the histone acetyltransferase General Control Non-derepressible 5 (TaGCN5) to *TaECR* promoters, leading to the epigenetic activation of cuticular wax biosynthesis [74]. Identifying interacting proteins of MYB transcription factors TaMYB60-1 and TaMYB60-2 could contribute to our understanding of transcriptional regulation of cuticular wax biosynthesis governed by MYB60 transcription factors.

Cuticular wax mixtures shield plant tissues from challenging environments and greatly contribute to plant adaptation to abiotic and biotic stresses. As extensively discussed in prior reviews, cuticular wax-associated traits have been widely employed for the genetic improvement of agronomically important crops like bread wheat [79,80]. For instance, increased leaf wax n-alkane concentration has been indirectly selected in breeding efforts to improve crop production in winter wheat [81]. Genetically manipulating *TaMYB60-1*, *TaMYB60-2*, *TaFATB1*, and *TaFATB2* genes essential for wheat cuticular wax biosynthesis using CRISPR (clustered regularly interspaced short palindromic repeats)-based approaches could contribute to improving wheat resistance against environmental stresses like drought and extreme temperature.

## 4. Materials and Methods

### 4.1. Plant Materials and Growth Conditions

Wheat cultivar Yannong 999 was cultivated for the gene expression analysis, virus-induced gene silencing, GC–MS, and leaf cuticle permeability analysis, whereas *A. thaliana* ecotype Col-0 was employed for protoplast preparation and dual-luciferase reporter assay. After surface sterilization, four wheat seeds were planted in 300 mL pots containing nutritive medium and sterile soil (1:1, *v*:*v*), and wheat seedlings were grown in climate chambers under 16 h light/8 h dark, 20 °C/18 °C day/night cycle, and 70% relative humidity. Arabidopsis seeds were planted in 200 mL plastic pots containing a commercial soil mix and grown in climate chambers at 22 °C and 70% RH under a 16 h light/8 h dark photoperiod.

### 4.2. Gene Expression Analysis

Expression levels of *TaMYB60-1*, *TaMYB60-2*, *TaFATB1*, or *TaFATB2* were analyzed using qRT-PCR, as previously described [71,75]. Briefly, total RNA was extracted from wheat BSMV-VIGS leaves using the TRIzol Reagent (Invitrogen, Carlsbad, CA, USA). After quality examination and genome DNA removal, 2 μg of total RNA was used to generate the cDNA that was employed as a template in the subsequent real-time PCR assay. Gene-specific primers were designed to analyze the expression levels of *TaMYB60-1*, *TaMYB60-2*, *TaFATB1*, or *TaFATB2*, and these primers are listed in Table S1. The qRT-PCR assay was repeated in three independent biological replicates. 

### 4.3. Virus-Induced Gene Silencing Assay

For the barley stripe mosaic virus-induced gene silencing assay, antisense (*as*) fragments of *TaMYB60-1*, *TaMYB60-2*, *TaFATB1*, or *TaFATB2* were amplified using primers listed in Table S1, and PCR products were employed for the generating constructs of the BSMV-*TaMYB60-1as*, BSMV-*TaMYB60-2as*, BSMV-*TaFATB1as*, or BSMV-*TaFATB2as*. The BSMV-VIGS silencing assay indicated genes were performing as previously described [82]. The newly grown wheat leaves with BSMV virus symptoms about two weeks post-BSMV infection were collected and subjected to gene expression analysis, as well as GC–MS analysis of cuticular wax accumulation. BSMV infection was reported to fail in changing the wax deposition and composition significantly [74]. The virus-induced gene silencing assay was repeated in three independent biological replicates with similar results.

### 4.4. Wheat Leaf Cuticle Permeability Measurement

Wheat leaf cuticle permeability was analyzed via water loss and chlorophyll leaching assay, as previously described [71,75]. Wheat plants were dipped in ultrapure water for 1 h in the dark to maintain stomatal closure, and the leaves were detached. For the water loss rate analysis, weights of detached leaves were measured every 1 h for 12 h. For the chlorophyll leaching assay, chlorophyll was extracted with 80% ethanol and measured with a spectrophotometer (TU1900, Shanghai Cany Precision Instrument, Shanghai, China) every 1 h for 12 h. The wheat leaf cuticle permeability analysis was repeated in three independent biological replicates.

### 4.5. GC–MS Analysis of Cuticular Wax Accumulation

The cuticular wax in wheat leaves was analyzed via GC–MS assay, as previously described [71,75]. Cuticular wax was first extracted with chloroform (Merck, Rahway, NJ, USA), derivatized by reaction with bis-N,O-trimethylsilyl trifluoroacetamide, and analyzed using a capillary GC (5890 Series II, Agilent Technologies, Santa Clara, CA, USA) and a flame ionization detector (FID) (6890 N, Agilent Technologies, Santa Clara, CA, USA) with a mass spectrometer (MSD 5973, Agilent Technologies, Santa Clara, CA, USA). Wax components were quantified based on FID peak areas relative to the internal standard n-Tetracosane (Merck, Rahway, NJ, USA). The GC–MS analysis of cuticular wax accumulation was repeated in three independent biological replicates. 

### 4.6. Dual-Luciferase Reporter Assay

For transcriptional activation analysis, the coding regions of AtMYB60, TaMYB60-1-5A, TaMYB60-1-5B, TaMYB60-1-5D, TaMYB60-2-4A, TaMYB60-2-4B and TaMYB60-2-4D and promoter regions of *TaFATB1-7A*, *TaFATB1-7D*, *TaFATB1-4A*, *TaFATB2-7A*, *TaFATB2-7B*, *TaFATB2-7D*, *TaCER1-6A* and *TaCER1-6B* were amplified using the primers listed in Table S1 and cloned into the vectors pRT-DBD and 5XGAL4-LUC, respectively. TaCER1 promoter sequences were reported by a previous study [78]. Dual-luciferase reporter assay measuring the transcriptional activation activity of TaMYB60-1 and TaMYB60-2 in *Arabidopsis* protoplast cells was performed, as described previously [71,75]. Arabidopsis leaf mesophyll protoplasts were isolated and transfected with indicated reporter and effector constructs. About 48 h post-protoplast transfection, the reporter luciferase activity (LucA) was analyzed and the Gal4 DNA-binding domain (DBD) was used to determine the basal LUC activity. The dual-luciferase reporter assay was repeated in three independent biological replicates.

## 5. Conclusions

In this study, partially redundant transcription factors TaMYB60-1 and TaMYB60-2 were identified as positive regulators of wheat cuticular wax biosynthesis. Knock-down of wheat *TaMYB60-1* and *TaMYB60-2* genes by virus-induced gene silencing resulted in attenuated wax accumulation and potentiated cuticle permeability. Furthermore, functions of TaFATB1 and TaFATB2, wheat homologs of Arabidopsis fatty acyl-ACP thioesterase AtFATB in cuticular wax biosynthesis were characterized. Silencing wheat *TaFATB1* and *TaFATB2* genes led to reduced wax accumulation and enhanced cuticle permeability, suggesting that *TaFATB1* and *TaFATB2* genes positively contribute to wheat cuticular wax biosynthesis. In addition, we demonstrated that TaMYB60-1 and TaMYB60-2 exhibit transcriptional activation ability and could directly activate the expression of wax biosynthesis genes *TaFATB1*, *TaFATB2* and *TaCER1*. These results support that transcription factor TaMYB60 stimulates wheat cuticular wax biosynthesis probably by activating the transcription of wax biosynthesis genes *TaFATB1*, *TaFATB2* and *TaCER1*. As a protective layer, wax mixtures shield plant tissues from environmental stresses, and wax-associated traits like increased leaf wax alkane concentrations have been indirectly selected in breeding efforts to improve wheat production. Therefore, this finding not only expands our understanding of transcriptional regulation of wheat cuticular wax biosynthesis, but also contributes to improving wheat against environmental stress like water deficit.

## Data Availability

Data presented here are available on request from correspondence.

## Supplementary Materials

The following supporting information can be downloaded at: https://www.mdpi.com/article/10.3390/ijms251910335/s1.
