# Supplementary material for: Wheat Transcription Factor TaMYB60 Modulates Cuticular Wax Biosynthesis by Activating TaFATB and TaCER1 Expression
Source: Int J Mol Sci. 2024 Sep 26;25(19):10335. doi: 10.3390/ijms251910335 (PMC11477597; doi:10.3390/ijms251910335)
Supplement: Supplementary file 1 [file ijms-25-10335-s001.zip › ijms-3202306-supplementary.pdf]

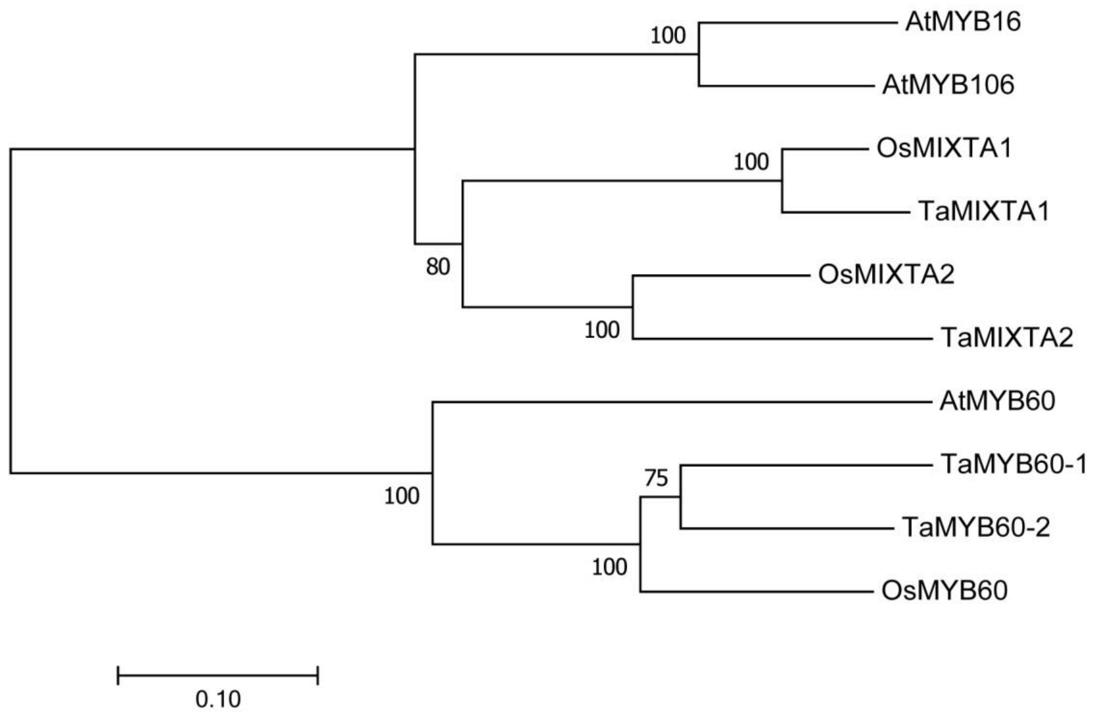


**Figure S1 Phylogenetic relationships of the MYB60, MYB16 and MYB106 homologs from Arabidopsis, rice, and bread wheat.** The phylogenetic tree was constructed using the maximum-likelihood method with 1000 bootstraps. Two letter genus-species prefixes: *At*, *Arabidopsis thaliana*; *Os*, *Oryza sativa*; *Ta*, *Triticum aestivum*.

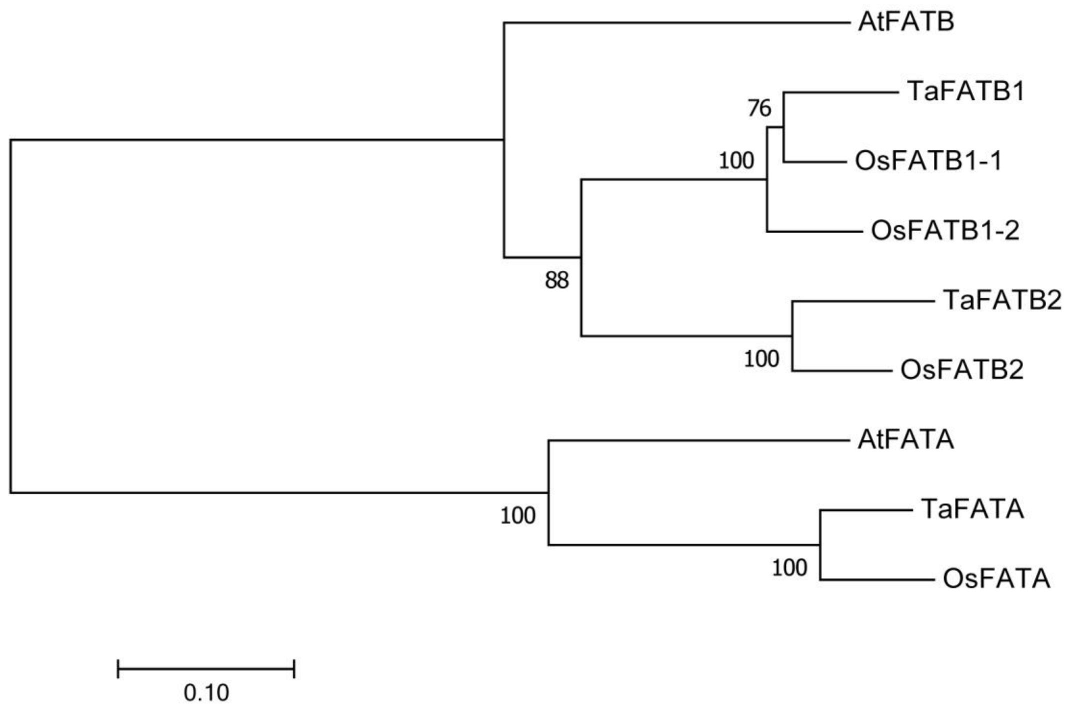


**Figure S2 Phylogenetic relationships of the FATA and FATB homologs from Arabidopsis, rice, and bread wheat.** The phylogenetic tree was constructed using the maximum-likelihood method with 1000 bootstraps. Two letter genus-species prefixes: *At*, *Arabidopsis thaliana*; *Os*, *Oryza sativa*; *Ta*, *Triticum aestivum*.

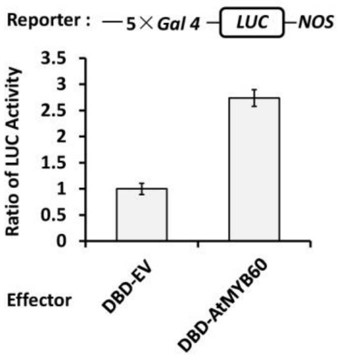


**Figure S3 Transcriptional activation ability of Arabidopsis transcription factor AtMYB60 measured in Arabidopsis protoplast cells.**

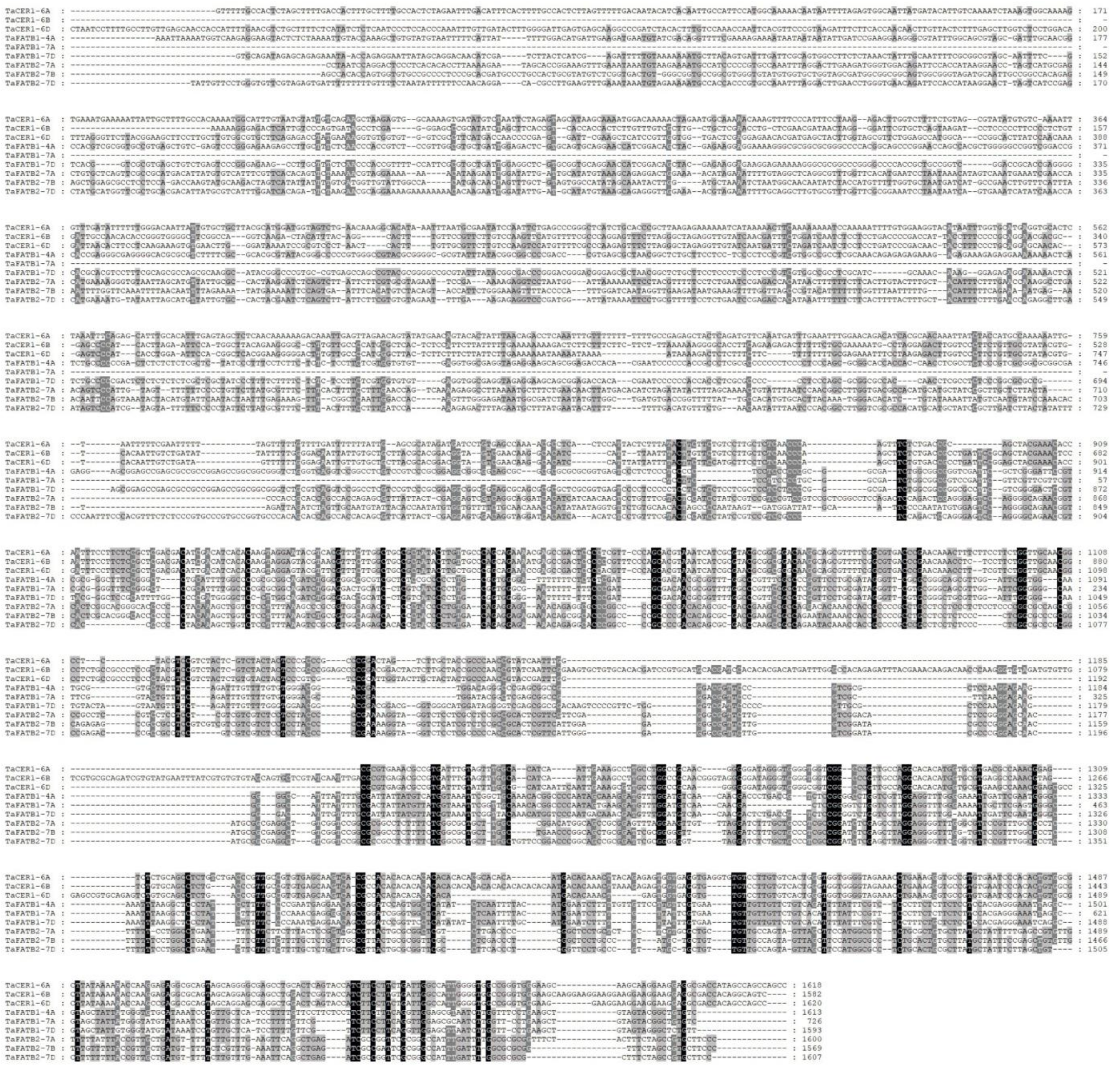

**Figure S4 Nucleotide sequence alignment of promoter regions in the constructs in Fig5C.** Identical nucleotide sequences among 9 promoter regions are shaded in dark, while nucleotide sequences conserved in at least 5 of the 9 promoter regions are shaded in gray.

**Supplemental Table S1. Primers used in this study**

| Primer Name | Sequence | Annotation |
|---|---|---|
| qRT-PCR-*TaMYB60-1*-F | 5' CCCTCCAGCTCCGCCGAG3’ | qRT-PCR primer for *TaMYB60-1*, F primer |
| qRT-PCR-*TaMYB60-1*-R | 5' GCGCATGGTGATCGTCGT3’ | qRT-PCR primer for *TaMYB60-1*, R primer |
| qRT-PCR-*TaMYB60-2*-F | 5' GGCGGTCTGTGCCGGTGA3' | qRT-PCR primer for *TaMYB60-2*, F primer |
| qRT-PCR-*TaMYB60-2*-R | 5' GTGGTTGTGGTGGTCGAA3' | qRT-PCR primer for *TaMYB60-2*, R primer |
| qRT-PCR-*TaFATB1*-F | 5' CTCGGGGTTCTTCCCCAC3' | qRT-PCR primer for *TaFATB1*, F primer |
| qRT-PCR-*TaFATB1*-R | 5' TTGAGGTTAACCTTAGAG3' | qRT-PCR primer for *TaFATB1*, R primer |
| qRT-PCR-*TaFATB2*-F | 5' CCGCCCAAGAGCGCCCTG3' | qRT-PCR primer for *TaFATB2*, F primer |
| qRT-PCR-*TaFATB2*-R | 5' TAGAACGTCCTCGGCTGC3' | qRT-PCR primer for *TaFATB2*, R primer |
| qRT-PCR-*TaCER1*-F | 5' CGGGGCCTTTGACTGAATG3' | qRT-PCR primer for *TaCER1*, F primer |
| qRT-PCR-*TaCER1*-R | 5' GAAGACGATCTGGTCGTC3' | qRT-PCR primer for *TaCER1*, R primer |
| pCa-*TaMYB60-1as*-F | 5'AAGGAAGTTTAAGTGGCAGCTGATGATGGA3' | For construct of BSMV-*TaMYB60-1as*, F primer |
| pCa-*TaMYB60-1as*-R | 5'AACCACCACCACCGTCGCCACCGACGTCAAACC3' | For construct of BSMV-*TaMYB60-1as*, R primer |
| pCa-*TaMYB60-2as*-F | 5'AAGGAAGTTTAAATGGCGCCTGATGCTGCTG3' | For construct of BSMV-*TaMYB60-2as*, F primer |
| pCa-*TaMYB60-2as*-R | 5'AACCACCACCACCGTTCAACTACGACGCCGACGA3' | For construct of BSMV-*TaMYB60-2as*, R primer |
| pCa-*TaFATB1as*-F | 5'AAGGAAGTTTAACGAGGTTTCCTTCGCCAT3' | For construct of BSMV-*TaFATB1as*, F primer |
| pCa-*TaFATB1as*-R | 5'AACCACCACCACCGTAGTGCCCCGATTTCAATA' | For construct of BSMV-*TaFATB1as*, R primer |
| pCa-*TaFATB2as*-F | 5'AAGGAAGTTTAACCGCCGTTTCCTGGGACT3' | For construct of BSMV-*TaFATB2as*, F primer |
| pCa-*TaFATB2as*-R | 5'AACCACCACCACCGTGAACTGGCGAGCATAGTC' | For construct of BSMV-*TaFATB2as*, R primer |
| pENTRY-*proTaFATB1-4A*-F | 5'GGGGACAAGTTTGTACAAAAAAGCAGGCTTCAAATTAAAATGGTCAAGAG3' | For the construction of pENTRY-*proTaFATB1-4A*, F primer |
| pENTRY-*proTaFATB1-4A*-R | 5'GGGGACCACTTTGTACAAGAAAGCTGGGTCGACACAGCCGTACTACAGC3' | For the construction of pENTRY-*proTaFATB1-4A*, R primer |
| pENTRY-*proTaFATB1-7A*-F | 5'GGGGACAAGTTTGTACAAAAAAGCAGGCTTCCTCCTTCTCCTTCCCCTGC3' | For the construction of pENTRY-*proTaFATB1-7A*, F primer |
| pENTRY-*proTaFATB1-7A*-R | 5'GGGGACCACTTTGTACAAGAAAGCTGGGTCGACACAGCCGTACTACAGC3' | For the construction of pENTRY-*proTaFATB1-7A*, R primer |
| pENTRY-*proTaFATB1-7D*-F | 5'GGGGACAAGTTTGTACAAAAAAGCAGGCTTCGTGCAGATAGAGCAGAGAA3' | For the construction of pENTRY-*proTaFATB1-7D*, F primer |
| pENTRY-*proTaFATB1-7D*-R | 5'GGGGACCACTTTGTACAAGAAAGCTGGGTCAACAGAGCCCTACTACAGC3' | For the construction of pENTRY-*proTaFATB1-7D*, R primer |
| pENTRY-*proTaFATB2-7A*-F | 5'GGGGACAAGTTTGTACAAAAAAGCAGGCTTCCCTAATCCAGGACTCCCTC3' | For the construction of pENTRY-*proTaFATB2-7A*, F primer |
| pENTRY-*proTaFATB2-7A*-R | 5'GGGGACCACTTTGTACAAGAAAGCTGGGT | For the construction of pENTRY-*proTaFATB2-7A*, R |

| | | |
|---|---|---|
| | CGGGAAGCACGGCTAGAAAG3' | primer |
| pENTRY-*proTaFATB2-7B*-F | 5'GGGGACAAGTTTGTACAAAAAAGCAGGCTTCAGCCACACCAGTGGTGTGC3' | For the construction of pENTRY-*proTaFATB2-7B*, F primer |
| pENTRY-*proTaFATB2-7B*-R | 5'GGGGACCACTTTGTACAAGAAAGCTGGGTCGGGAAGCACGGCTAGAAAC3' | For the construction of pENTRY-*proTaFATB2-7B*, R primer |
| pENTRY-*proTaFATB2-7D*-F | 5'GGGGACAAGTTTGTACAAAAAAGCAGGCTTCTATTGTTCCTGGGTGTTCG3' | For the construction of pENTRY-*proTaFATB2-7D*, F primer |
| pENTRY-*proTaFATB2-7D*-R | 5'GGGGACCACTTTGTACAAGAAAGCTGGGTCGGAAGCACGGCTAGAAAGC3' | For the construction of pENTRY-*proTaFATB2-7D*, R primer |
| pENTRY-*proTaCER1-6A*-F | 5'GGGGACAAGTTTGTACAAAAAAGCAGGCTTCGTTTTTGCCACTCTAGCTT3 ' | For the construction of pENTRY-*proTaCER1-6A*, F primer |
| pENTRY-*proTaCER1-6A*-R | 5'GGGGACCACTTTGTACAAGAAAGCTGGGTCGGCTGGCTGGCTATGGTCGC3' | For the construction of pENTRY-*proTaCER1-6A*, R primer |
| pENTRY-*proTaCER1-6B*-F | 5'GGGGACAAGTTTGTACAAAAAAGCAGGCTTCAAAAGGGAGACTCATTGTC3' | For the construction of pENTRY-*proTaCER1-6B*, F primer |
| pENTRY-*proTaCER1-6B*-R | 5'GGGGACCACTTTGTACAAGAAAGCTGGGTCGACTGCCTGTGGTCGCCTC3' | For the construction of pENTRY-*proTaCER1-6B*, R primer |
| pENTRY-*proTaCER1-6D*-F | 5'GGGGACAAGTTTGTACAAAAAAGCAGGCTTCCTAATCCTTTTGCCTTGTTG3' | For the construction of pENTRY-*proTaCER1-6D*, F primer |
| pENTRY-*proTaCER1-6D*-R | 5'GGGGACCACTTTGTACAAGAAAGCTGGGTCGGCTGGCTGTGGTCGCCTC3' | For the construction of pENTRY-*proTaCER1-6D*, R primer |
| pENTRY-*AtMYB60*-F | 5'GGGGACAAGTTTGTACAAAAAAGCAGGCTTCATGGGTAGGCCTCCATGCT3 ' | For the construction of pENTRY-*AtMYB60*, F primer |
| pENTRY-*AtMYB60*-R | 5'GGGGACCACTTTGTACAAGAAAGCTGGGTCTTAAAGCATATTAGAGAGC3' | For the construction of pENTRY-*AtMYB60*, R primer |
| pENTRY-*TaMYB60-1-5A*-F | 5'GGGGACAAGTTTGTACAAAAAAGCAGGCTTCATGGGGAGGCCGCCGTGCTG3' | For the construction of pENTRY-*TaMYB60-1-5A*, F primer |
| pENTRY-*TaMYB60-1-5A*-R | 5'GGGGACCACTTTGTACAAGAAAGCTGGGTCTAGTACAGCAATGGCAGG3' | For the construction of pENTRY-*TaMYB60-1-5A*, R primer |
| pENTRY-*TaMYB60-1-5B*-F | 5'GGGGACAAGTTTGTACAAAAAAGCAGGCTTCATGGGGAGGCCGCCGTGCTG3 ' | For the construction of pENTRY-*TaMYB60-1-5B*, F primer |
| pENTRY-*TaMYB60-1-5B*-R | 5'GGGGACCACTTTGTACAAGAAAGCTGGGTCCTAGTACAGCAATGGCAGG3' | For the construction of pENTRY-*TaMYB60-1-5B*, R primer |
| pENTRY-*TaMYB60-1-5D*-F | 5'GGGGACAAGTTTGTACAAAAAAGCAGGCTTCATGGGGAGGCCGCCGTGCTG3' | For the construction of pENTRY-*TaMYB60-1-5D*, F primer |
| pENTRY-*TaMYB60-1-5D*-R | 5'GGGGACCACTTTGTACAAGAAAGCTGGGTCCTAGTACAGCAATGGCAGG3' | For the construction of pENTRY-*TaMYB60-1-5D*, R primer |
| pENTRY-*TaMYB60-2-4A*-F | 5'GGGGACAAGTTTGTACAAAAAAGCAGGCTTCATGGGGAGGCCGCCGTGCTG3' | For the construction of pENTRY-*TaMYB60-2-4A*, F primer |
| pENTRY-*TaMYB60-2-4A*-R | 5'GGGGACCACTTTGTACAAGAAAGCTGGGTCCTAGAACAGCATTGGAACG3' | For the construction of pENTRY-*TaMYB60-2-4A*, R primer |
| pENTRY-*TaMYB60-2-4B*-F | 5'GGGGACAAGTTTGTACAAAAAAGCAGGCTTCATGGGGAGGCCGCCGTGCTG3' | For the construction of pENTRY-*TaMYB60-2-4B*, F primer |

| | | |
|---|---|---|
| pENTRY-*TaMYB60-2-4B*-R | 5'GGGGACCACTTTGTACAAGAAAGCTGGGTCCTAGAACAGCATTGGAACG3' | For the construction of pENTRY-*TaMYB60-2-4B*, R primer |
| pENTRY-*TaMYB60-2-4D*-F | 5'GGGGACAAGTTTGTACAAAAAAGCAGGCTTCATGGGGAGGCCGCCGTGCTG3' | For the construction of pENTRY-*TaMYB60-2-4D*, F primer |
| pENTRY-*TaMYB60-2-4D*-R | 5'GGGGACCACTTTGTACAAGAAAGCTGGGTCCTAGAACAGCATTGGAACG3' | For the construction of pENTRY-*TaMYB60-2-4D*, R primer |
